# Molecular epidemiology of hepatitis E virus infections in Shanghai, China

**DOI:** 10.1186/1743-422X-8-541

**Published:** 2011-12-15

**Authors:** Yumin Zhu, Fusheng Si, Dianjun Cao, Xiaoming Yu, Ruisong Yu, Shijuan Dong, Fenfen Huang, Yuanshu Zhang, Zhen Li

**Affiliations:** 1 Institute of Animal Science and Veterinary Medicine, Shanghai Academy of Agricultural Sciences, Shanghai 201106, China; 2 Shanghai Key Laboratory of Agricultural Genetics and Breeding, Shanghai 201106, China; 3 Nanjing Agricultural University, Nanjing, Jiangsu 210095, China; 4 Center for Molecular Medicine and Infectious Diseases, Department of Biomedical Sciences and Pathobiology, College of Veterinary Medicine, Virginia Polytechnic Institute and State University, Blacksburg, VA 24061-0913, USA; 5 Jiangxi Agricultural University, Nanchang, Jiangxi 330045, China

**Keywords:** Hepatitis E virus, Epidemiology, Shanghai municipality, Virus genotypes, Virus subtypes

## Abstract

**Background:**

Hepatitis E virus (HEV) causes acute or fulminant hepatitis in humans and is an important public health concern in many developing countries. China has a high incidence of HEV epidemics, with at least three genotypes (1, 3 and 4) and nine subtypes (1b, 1c, 3b, 4a, 4b, 4d, 4g, 4h and 4i) so far identified. Since genotype 3 and the newly identified subtype 4i have been exclusively limited geographically to Shanghai and its neighboring provinces, the epidemiology of HEV infections within the municipality, a major industrial and commercial center, deserves closer attention.

**Findings:**

A total of 65 sequences, 60 located within the HEV SH-SW-zs1 genome [GenBank:EF570133], together with five full-length swine and human HEV genomic sequences, all emanating from Shanghai, were retrieved from GenBank. Consistent with the primary role of genotype 4 in China overall, analysis of the sequences revealed this to have been the dominant genotype (58/65) in Shanghai. Six HEV subtypes (3b, 4a, 4b, 4d, 4h and 4i) were also represented. However, although subtype 4a is the dominant subtype throughout China, subtype 4i (29/65) was the most prevalent subtype among the Shanghai sequences, followed by subtypes 4d (10/65) and 4h (9/65). Subtypes 4h, 4i and 4d were found in both swine and humans, whereas 4b was found only in swine and subtype 4a only in humans.

**Conclusions:**

Six different swine and human HEV subtypes have so far been documented in Shanghai. More molecular epidemiological investigations of HEV in swine, and particularly among the human population, should be undertaken.

## Findings

Hepatitis E virus (HEV), the causative agent of acute or fulminant hepatitis in humans, is an important public health concern in many developing countries. It is estimated that about two billion people, or one-third of the world population, live in areas where HEV is endemic and are therefore at risk of infection [1]. The disease is thought to be transmitted by the fecal-oral route, usually through contaminated drinking water.

HEV is a non-enveloped, single stranded, positive-sense RNA virus belonging to the family *Hepeviridae*. At least four mammalian HEV genotypes have been recognized [2]. Genotypes 1 and 2 are primarily associated with fecal-oral transmission among humans and, in developing countries, can lead to waterborne jaundice epidemics. Genotypes 3 and 4 circulate in humans and several animal species, and are associated with sporadic infections among humans in industrialized countries [3]. In addition, two putative HEV genotypes, one from the Norway rat (*Rattus norvegicus*) [4] and the other from wild boar [5], were recently reported.

A recent study in China, where there is a high frequency of HEV epidemics, has shown that HEV seroprevalence among the general population was almost 40% and increased with age at a rate of about 1% per year [6]. Furthermore, the number of fecal samples taken from young swine that tested positive for HEV RNA ranged between 20-48% [7,8]. According to Zhu *et al.*[9], at least three genotypes (1, 3 and 4) and nine subtypes (1b, 1c, 3b, 4a, 4b, 4d, 4g, 4h and 4i) of human and swine HEV have so far been documented in China. Since genotype 3 and the newly identified subtype 4i have been limited geographically exclusively to Shanghai and its neighboring provinces, the municipality, as a major industrial and commercial center, is deserving of close attention with respect to HEV infection epidemiology.

**Figure 1:**
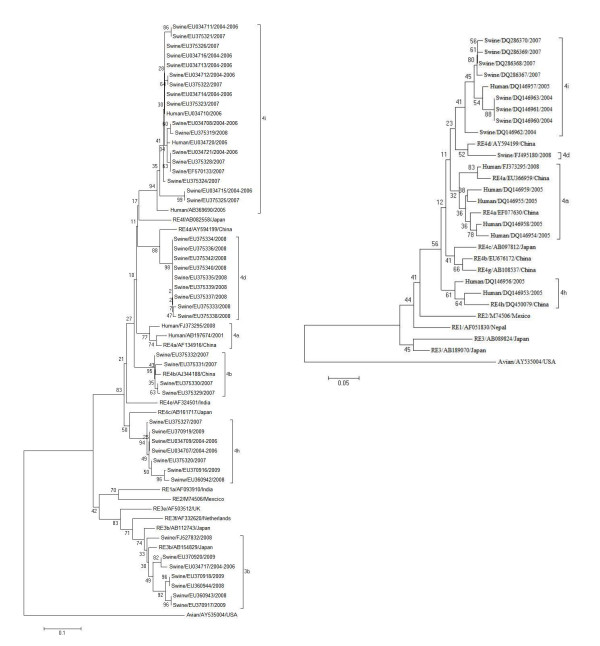
**Phylogenetic tree depicting genotypic/subgenotypic status of swine and human HEV strains isolated in Shanghai**. A) Analyses based on 5 full-length swine and human HEV genomic sequences and 44 ORF2 sequences located between nt 6,104 and nt 6,256 within the HEV SH-SW-zs1 genome [GenBank:EF570133]. B) Analyses based on 16 sequences located between nt 6,360 and nt 6,509 within the HEV SH-SW-zs1 genome. Percent bootstrap support is indicated at each node. For each phylogeny, HEV subtypes were indicated on the outside of the square brackets that define the HEV subtypes. Each branch is labeled with the host of the HEV isolate, GenBank accession number and the year the strain was isolated. Reference sequences are labeled with the prefix RE, the subtype of the sequence, the GenBank accession number and the geographical source.

A total of 60 sequences located within the HEV SH-SW-zs1 genome (GenBank: EF570133] (44 between nt 6104 and nt 6256, and 16 between nt 6360 and nt 6509) together with five full-length swine and human HEV genomic sequences, all derived from Shanghai, were retrieved from GenBank as of June, 2011. All the sequences were aligned with Clustal W (version 1.8), and the sequence percent identity was calculated using Lasergene (version 7.10; DNAstar). Phylogenetic trees were constructed by the neighbor-joining method [10], based on the partial nucleotide sequences of the ORF2 region. Bootstrap values were determined on 3,000 re-samplings of the data sets [11]. The criteria used to define HEV genotypes or subtypes were adopted from Lu *et al*. [12]. These authors demonstrated that an assemblage of 300-450 nucleotides at the 5' end of the HEV ORF2 region was highly conserved and that phylogenetic analysis based on this region provided accurate information about the genetic relationships between the HEV isolates and their evolutionary state. The accession numbers of HEV reference sequences are shown in Figure 1

Phylogenetic analysis indicated that six different HEV subtypes, i.e. 3b, 4a, 4b, 4d, 4h and 4i (Figure 1) have so far been prevalent in Shanghai. Genotype 4 was the most highly represented genotype among the 65 Shanghai sequences, and subtype 4i (29/65), followed by 4d (10/65) and 4h (9/65), the most prevalent subtypes. Of the 29 sequences classified as subtype 4i, four were of human origin and 25 were from swine. HEV strain SH-SW-zs1 [GenBank: EF570133], originating from swine and regarded as the Shanghai prototype HEV strain, belonged to subtype 4i. Furthermore, all the subtype 4i HEV strains so far reported in China were isolated in the eastern part of the country, with 70% emanating from Shanghai, suggesting that subtype 4i strains are perhaps indigenous to the municipality. Also included among this subtype was the HEV strain, E067-SIJ05C [GenBank: AB369690], collected from a patient suffering from acute hepatitis E in Japan who had traveled to Shanghai before the onset of the disease. Furthermore, the partial sequence [GenBank: EU034710] of another subtype 4i strain isolated from a human source in Shanghai was found to be virtually identical to a partial HEV sequence [GenBank: EU034714] obtained from a sample of swine serum in eastern China during the same period, suggesting that the isolates had a common origin [13]. To date, a total of nine subtype 4i HEV strains identified from full genomic sequences deposited in GenBank have emanated either from Japan or from Shanghai and neighboring Jiangsu Province. HEV strains of human origin emanating from Japan (JYN-Shiz08L and JKS-Shiz07L) and from Shanghai (E067-SIJ05C) were closely related to HEV strains isolated from wild boar (wbJGF08-1) [14,15] and from swine (SAAS-FX17) [16], respectively. In addition, when phylogenetic clustering of subtype 4i HEV strains was examined (data not shown), sequences from Japan and Shanghai were positioned on different subtype 4i branches, suggesting that each of these two groups of strains derived from different origins. When compared with other HEV subtype 4 strains across the whole genome, only two specific amino acid substitutions were identified within the ORF1 of subtype 4i. One substitution was located in the methyltransferase motif and the other in the macro domain and might therefore influence HEV replication [17].

In this study, only subtypes 4h, 4i and 4d were found in both swine and humans, whereas subtypes 4a and 4b were confined to either human or swine populations, respectively. According to Zhu *et al*. [16], subtypes 4a and 4b HEV were capable of infecting both humans and swine. Subtype 4a HEV is widely distributed among both the swine and human populations in China as a whole but swine subtype 4a HEV has not been identified in Shanghai. A human subtype 4a strain isolated in Shanghai, JYI-ChiSai01C [GenBank: AB197674], showed the highest nucleotide similarity (94.0%) with a swine HEV strain Ch-S-1, from Jilin Province [GenBank: EF077630], suggesting that 4a subtypes in Shanghai were also zoonotic. Human subtype 4b HEV has a more limited distribution in China than the swine counterpart [16] and, so far, no human subtype 4b strain has been reported in Shanghai. However, not every sample from hepatitis E cases in Shanghai has been analyzed sufficiently and the possibility that human subtype 4b exists among the population of the municipality cannot be excluded.

Genotype 3 HEV strains were geographically limited to Shanghai and neighboring provinces, and seven sequences were classified into subtype 3b. No human genotype 3 HEV sequences were among those from Shanghai although one strain, EChN22 [GenBank:HM439285], was reported recently in neighboring Jiangsu Province [18]. The EChN22 isolate clustered closely with Shanghai swine isolate FJ527832 and shared 97.2% nucleotide and 99.6% amino acid homologies, which suggested that genotype 3 strains prevalent in Shanghai possibly participated in human-swine transmission.

No genotype 1 HEV representatives were found in this study, possibly due to the limited number (10/65) of human HEV sequences available.

In summary, six different HEV subtypes, i.e. 3b, 4a, 4b, 4d, 4h and 4i, have so far been shown to be prevalent in Shanghai, with genotype 4 and subtype 4i the dominant forms. Only subtypes 4h, 4i and 4d were found in both swine and human hosts, whereas subtypes 4a and 4b were confined to either human or swine populations, respectively. A recent epidemiological investigation has shown that the average incidence of HEV RNA positives among the swine population of Shanghai was approximately 20.0%, and that serum IgG positives reached 72.18% [19]. Hepatitis infection rates (all types) ranged between 15.0-35.0% among the younger population and reached 47.0% in older people. Furthermore, 39.4-69.7% cases of sporadic hepatitis E involved co-infection with other hepatitis viruses [20]. Human hepatitis E infections have increased significantly in recent years and, in Shanghai, have now become the third most likely cause of hepatitis disease after hepatitis A and B [21]. Consequently, more molecular epidemiology investigations of HEV among swine, and particularly human, populations should be undertaken to assist in HEV control and prevention.

## List of abbreviations

HEV: Hepatitis E virus; ORF: Open reading frame

## Competing interests

The authors declare that they have no competing interests.

## Authors' contributions

YMZ, FYS, RSY, YSZ and ZL participated in the study design, YMZ, SJD and FSS performed the sequence analysis, and all authors participated in writing and revising the manuscript. All authors have read and approved the final version.

## References

[B1] Chandra VTS Kalia M Jameel S Molecular biology and pathogenesis of hepatitis E virus J Biosci 2008 33 451 464 10.1007/s12038-008-0064-1 19208971

[B2] Emerson SU Anderson D Arankalle A Meng XJ Purdy M Schlauder GG Tsarev SA Fauquet CM, Mayo MA, Maniloff J, Desselberger U, Ball LA Hepevirus Virus taxonomy VIIIth report of the ICTV 2004 London:Elsevier/Academic Press 851 855

[B3] Teo CG The two clinico-epidemiological forms of hepatitis E J Viral Hepat 2007 14 295 297 10.1111/j.1365-2893.2007.00857.x 17439517

[B4] Johne R Plenge-Bönig A Hess M Ulrich RG Reetz J Schielke A Detection of a novel hepatitis E-like virus in faeces of wild rats using a nested broad-spectrum RT-PCR J Gen Virol 2010 91 750 758 10.1099/vir.0.016584-0 19889929

[B5] Takahashi M Nishizawa T Sato H Sato Y Jirintai Nagashima S Okamoto H Analysis of the full-length genome of a hepatitis E virus isolate obtained from a wild boar in Japan that is classifiable into a novel genotype J Gen Virol 2011 92 902 908 10.1099/vir.0.029470-0 21228128

[B6] Li RC Ge SX Li YP Zheng YJ Nong Y Guo QS Zhang J Ng MH Xia NS Seroprevalence of hepatitis E virus infection, rural southern People's Republic of China Emerg Infect Dis 2006 12 1682 1688 10.3201/eid1211.060332 17283617 PMC3372335

[B7] Li Z Yu SS Dong SJ Zhu YM Si FS Shen SY Jiang ZQ Yu RS Zou SX Reduced prevalence of genotype 3 HEV in Shanghai pig farms and hypothetical homeostasis of porcine HEV reservoir Vet Microbiol 2009 137 184 189 10.1016/j.vetmic.2008.12.013 19150181

[B8] Geng J Wang L Wang X Fu H Bu Q Liu P Zhu Y Wang M Sui Y Zhuang H Potential risk of zoonotic transmission from young swine to human: seroepidemiological and genetic characterization of hepatitis E virus in human and various animals in Beijing, China J Viral Hepat 2011 18 e583 590 10.1111/j.1365-2893.2011.01472.x 21914080

[B9] Zhu YM Dong SJ Si FS Yu RS Yu XM Zou SX Li Z Swine and human hepatitis E virus (HEV) infection in China J Clin Virol 2011 52 155 157 10.1016/j.jcv.2011.06.023 21831705

[B10] Saitou N Nei M The neighbor-joining method: a new method for reconstructing phylogenetic trees Mol Biol Evol 1987 4 406 425 3447015 10.1093/oxfordjournals.molbev.a040454

[B11] Felsenstein J Confidence limits on phylogenies: an approach using the bootstrap Evolution 1985 783 791 28561359 10.1111/j.1558-5646.1985.tb00420.x

[B12] Lu L Li C Hagedorn CH Phylogenetic analysis of global hepatitis E virus sequences: genetic diversity, subtypes and zoonosis Rev Med Virol 2006 16 5 36 10.1002/rmv.482 16175650

[B13] Zhang W Shen Q Mou J Gong G Yang Z Cui L Zhu J Ju G Hua X Hepatitis E virus infection among domestic animals in eastern China Zoonoses Public Hlth 2008 55 291 298 10.1111/j.1863-2378.2008.01136.x 18638181

[B14] Sato Y Sato H Naka K Furuya S Tsukiji H Kitagawa K Sonoda Y Usui T Sakamoto H Yoshino S Shimizu Y Takahashi M Nagashima S Jirintai Nishizawa T Okamoto H A nationwide survey of hepatitis E virus (HEV) infection in wild boars in Japan: identification of boar HEV strains of genotypes 3 and 4 and unrecognized genotypes Arch Virol 2011 156 1345 1358 10.1007/s00705-011-0988-x 21475985

[B15] Takahashi K Kitajima N Abe N Mishiro S Complete or near-complete nucleotide sequences of hepatitis E virus genome recovered from a wild boar, a deer, and four patients who ate the deer Virology 2004 330 501 505 10.1016/j.virol.2004.10.006 15567444

[B16] Zhu YM Yu XM Si FS Zhang YS Zou SX Li Z Full-genome sequence analysis of a hepatitis E virus (HEV) strain SAAS-FX17 and potential disease severity determinants identification between genotype 3 and genotype 4 HEV strains Virology 2011 submitted

[B17] Ahmad I Holla RP Jameel S Molecular virology of hepatitis E virus Virus Res 2011 doi:10.1016/j.virusres.2011.02.011 10.1016/j.virusres.2011.02.011 PMC3130092 21345356

[B18] Yan Y Zhang W Shen Q Cui L Hua X Prevalence of four different subgenotypes of genotype 4 hepatitis E virus among swine in the Shanghai area of China Acta Vet Scand 2008 50 12 10.1186/1751-0147-50-12 18513433 PMC2426689

[B19] Zhou JP Sun QY Liu PH Xue X Li KH Lu J Zhang WY Qi XY Serological investigation on hepatitis E of several animal species in Shanghai Prog in Vet Med 2006 27 85 88 (Article in Chinese)

[B20] Li YT Zhu YY Shen WG Zhang AX Zhang JM Ren H Yuan GP Gu LJ The analysis of HEV genotypes isolated from sporadic acute hepatitis E in Shanghai Virologica Sinica 2002 17 106 109

[B21] Kang LY Pan QC Jin ZC Zhou TK Fang H Xue YL Zhang W Jiang Q Zhu QR Yao GB Zhang JM Zheng XH The molecular investigation of hepatitis C and E virus infection in Shanghai J Prev Med 1995 7 438 441

